# Dissertation on Preparation of a Cavity in a Tooth, Preparatory to Plugging

**Published:** 1846-09

**Authors:** W. H. Dwinelle


### ARTICLE III.

*Dissertation on Preparation of a Cavity in a Tooth, preparatory to Plugging*. By W. H. Dwinelle. Read before the American Society of Dental Surgeons, at its Seventh Annual Meeting, held in the City of New York, August 4th, 1846.

*Mr, President and Gentlemen :*

I am aware that I am about to propose a course of practice to you, wholly at variance with the present system, and which, at first, may perhaps meet with your unqualified disapprobation. And notwithstanding the gentle admonition which so naturally comes up, of "Let him that thinketh he standeth take heed," &c. still I feel so fortified, by experience and observation, in the position I am about to take, that I unshrinkingly press forward to its declaration.

In the operation of plugging teeth, we have ever deemed it important—indeed, indispensable—that every particle of decayed and discolored matter should be removed throughout the entire cavity of the tooth; nor, indeed, have we considered ourselves justified in regarding our operation as perfect, unless we had followed this course to the letter. That this course of practice is objectionable in the *general*, I am not so unblushing as to declare; but that there are circumstances wherein it is not only proper, but *duty*, to leave discolored and even softened parts in the tooth, I am prepared to declare and prove.

It is the experience of us all, that oftentimes, in removing decayed parts from the cavities of teeth, notwithstanding we are safely enabled to displace all decayed and discolored matter from all surrounding parts, still we find that the *nerve* and its immediate vicinity is overcapped and protected with discolored bone, which needs but little license to call decayed. To proceed and remove every particle of decay from the tooth, would be to uncover the nerve, and surround the operation with difficulties which I will not at this time enumerate.

Under circumstances like the foregoing, after having thoroughly cleaned the walls and such parts of the bottom of the cavity of the tooth as I can safely do without interfering with the nerve, I wash out the cavity with a solution of soda, and then proceed to fill the tooth in the usual manner, taking care, however, especially if the parts are much softened immediately over the nerve, to skilfully build an arch over that point, so as to enable it to resist the severe pressure of filling, finishing, &c.

Here, then, we have actual decay sealed up within the centre of the tooth, and that, too, in immediate contact with the nerve! If the first principle of the exploded theory of "internal caries" were true, I have most effectually insured the certain destruction of the tooth, and *malpractice* is but a poor name to distinguish my operation. But my observation and experience, as well as that of several of my professional friends, proves to the contrary. I have practised in the manner above described for the last four years, and I do not now recollect of a single instance of failure. I have a tooth in my head—literally a *golden* one—filled and treated as above by Dr. Westcott, nearly three years ago, as perfect as the day of its completion, and from which I have never experienced the least inconvenience.

In considering teeth treated in the manner described, the question very naturally presents itself, "*Does the decay go on?*" I answer, emphatically, *no.* The decay of the teeth is entirely a chemical action, and depends upon *external* agencies alone for its progress, such as air and water, and such other influences as will promote a constant acidulated and decomposing action. The *decay* possesses no quality in itself of advancement, and when the cavity within the tooth is so completely and skilfully closed, as to cut off *all* communication from *without*, it is as impossible for the decay to advance, as it would be if the nerve was capped with bone of the purest whiteness. I am inclined to think it matters but little if the bone that protects the nerve of a tooth *has* lost a portion of its lime, so long as it has sufficient strength to resist the force of a proper plugging. This discolored portion is but a modified character of real bone, a little more of gelatine and less of lime is all the difference.

The fact that the nerve is constantly receding with the age of the patient, to say the least, encourages our operation; and if our work is skilfully performed, and does not interfere with the internal membranes of the tooth, so as to cause inflammation, justifies us in the conclusion that it is permanent and complete.

The uncovering of the nerve of a tooth ever involves difficulties of a greater or less degree, and which oftentimes can only be overcome by the removal of the tooth itself. To expose the nerve is always to subject ourselves to the necessity of removing it: for our experience has been emphatic in its teachings to us, that it is ever unsafe to fill a tooth when the membrane of its nerve is broken. Inflammation, suppuration and destruction are a part of the leading features in the history of its consequences. When exposed, the removal of this delicate fibre is inevitable, and though at times it can be done with comparatively little pain, and generally with safety, yet at others, especially in back teeth, it is extremely painful and tedious.

True it is, that after its removal, by filling the cavity to its extremity with gold, we have performed a comparatively successful operation; but when we reflect that we have destroyed a vein, an artery and a nerve, all essential to the vitality and healthful existence of a tooth, we are driven to the conclusion, that our operation is far from being perfect, and we regret that we cannot with more certainty anticipate a more favorable result; we have but chosen a *lesser* evil, the least of two. I propose that we take the *least* evil, the least of *three*, the least of all!

